# Scale-dependent biases in systematic land cover maps undermine freshwater ecological assessment

**DOI:** 10.1007/s10661-026-15882-1

**Published:** 2026-09-14

**Authors:** Iñaki Fernández de Larrea, Jon González-Ibarzabal, Aitor Bastarrika, Aitor Larrañaga

**Affiliations:** 1 Department of Plant Biology and Ecology, Faculty of Science and Technology, University of the Basque Country (UPV/EHU), Bº Sarriena S/N, 48940 Leioa, Spain; 2 Built Heritage Research Group, University of the Basque Country (UPV/EHU), C/Nieves Cano, 12, 01006 Vitoria-Gasteiz, Spain; 3 Department of Mining and Metallurgical Engineering and Materials Science, School of Engineering of Vitoria-Gasteiz, University of the Basque Country (UPV/EHU), C/Nieves Cano, 12, 01006 Vitoria-Gasteiz, Spain

**Keywords:** Land cover change, Systematically produced maps, Remote sensing, Natura 2000, Freshwater ecosystems

## Abstract

Land cover is a key determinant of landscape structure and ecological processes across spatial and temporal scales. In freshwater ecosystems, where riparian and catchment land cover regulate hydrology, nutrient inputs, and habitat quality, most ecological assessments rely on systematically produced land cover maps whose reliability varies with scale and context. Despite their widespread use, the extent to which these products accurately represent landscape patterns and long-term dynamics at ecologically relevant scales remains poorly understood. Here, we evaluate the reliability of systematic land cover maps and assess whether Landsat-based supervised classification (SC) provides more accurate and temporally consistent representations of land cover patterns in draining catchments and riparian areas. We analysed five headwater catchments (< 70 km^2^) within Natura 2000 sites in northern Spain and reconstructed land cover dynamics over four decades (1984–2023) using Random Forest classification implemented in Google Earth Engine. SC results were compared with CORINE Land Cover, SIOSE, and the Spanish National Forest Inventory across three spatial scales, relevant for freshwater ecological processes: catchment, riparian corridor, and reach. SC achieved consistently high accuracy across all periods (mean overall accuracy = 87.8%), outperforming CORINE and SIOSE and matching NFI only for forest classes. Discrepancies between SC and systematic maps increased at finer spatial scales and varied with landscape context, particularly in heterogeneous riparian environments. Systematic maps also showed limited temporal consistency, frequently misrepresenting land cover trends relative to SC. These scale-dependent and context-specific biases can alter estimates of riparian vegetation and landscape composition and their temporal dynamics, potentially leading to misleading assessments of freshwater ecosystem condition. Our findings demonstrate that reliance on coarse-scale land cover products can distort ecological inference, particularly for freshwater ecosystems where ecological condition depends strongly on catchment and riparian landscape structure. Integrating reproducible, remote sensing–based classifications offers a practical pathway to improving the accuracy and interpretability of land cover indicators in landscape ecological research.

**Supplementary Information:**

The online version contains supplementary material available at https://doi.org/10.1007/s10661-026-15882-1.

## Introduction

Land cover shapes ecosystem structure and function across multiple spatial scales. At broad scales, it constrains species distributions by influencing dispersal and colonisation, thereby affecting community composition (Correia et al., 2021; Marta et al., 2021). At finer scales, land cover regulates energy and matter fluxes in terrestrial and aquatic systems, influencing processes such as nutrient cycling and organic matter inputs (Ehnes, 2019; Jonsson & Stenroth, 2016). Land cover change and land use intensification are among the leading global drivers of biodiversity loss (IPBES, 2019), interacting with climate change (Oliver & Morecroft, 2014) and local stressors such as nutrient enrichment and hydrological alteration (Segurado et al., 2018). Consequently, accurate land cover information is essential for ecological studies and conservation planning. Understanding ecosystem condition also requires historical land cover data, as many ecological processes operate with time lags that create legacy effects (Bürgi et al., 2017). These effects influence species richness and composition (Janssen et al., 2018), soil nutrient availability (Fraterrigo et al., 2005), and water quality in adjacent aquatic ecosystems (Martin et al., 2017), determining the success of restoration efforts (Verheyen, 2022). Accurate classification of present land cover and reliable reconstruction of present and past land cover are therefore critical for interpreting present ecological conditions and for assessing the status of freshwater ecosystems, whose functioning depends strongly on catchment and riparian landscape structure (Sweeney & Newbold, 2014; Szabolcs et al., 2022). However, the extent to which different land cover products reliably represent spatial and temporal landscape patterns remains poorly understood.

In practice, assessments of spatial and temporal land cover patterns rely heavily on systematically produced datasets developed by national and international agencies (Pandey et al., 2021). Widely used examples include CORINE Land Cover in Europe (Büttner et al., 2004), SIOSE in Spain (Bosque González et al., 2005), and thematic maps such as national forest inventories (Álvarez-González et al., 2014; Selwyn et al., 2024). These datasets are extensively used in ecological research, including habitat modelling, landscape connectivity analyses, and other indicators that support freshwater ecological assessment (Fernández et al., 2014; Lumia et al., 2023; Viña et al., 2008)—over 900 studies in ecology and biodiversity conservation have relied on CORINE, SIOSE, or NFI (Web of Science search, April 2025). Their large spatial extent, standardised classification schemes, and multi-temporal availability make them particularly attractive for analysing landscape patterns across large regions and long time periods. However, because systematically produced maps are designed primarily for broad-scale monitoring, their application to finer spatial grains introduces important limitations, as highlighted by recent reviews (Wang et al., 2023). Coarse minimum mapping units (e.g., 25 ha in CORINE), semantic inconsistencies among classification schemes, and methodological changes across versions can bias estimates of land cover composition and change (Catalá-Mateo et al., 2008; García Álvarez & Camacho Olmedo, 2023). These scale-related biases are especially pronounced in heterogeneous landscapes and in narrow spatial extents such as riparian corridors or stream reaches, where small differences in grain and extent can substantially alter inferred landscape patterns (Tormos et al., 2011). As a result, ecological indicators derived from these products may be misleading because scale-dependent biases can distort estimates of land-cover composition, temporal trends, and landscape patterns, complicating interpretation of ecosystem–landscape relationships.

The scale-related limitations of systematically produced maps highlight the need for approaches that can capture land cover patterns more accurately and consistently. Advances in remote sensing offer alternatives for generating land cover representations with higher spatial and temporal consistency, which is essential for interpreting ecological patterns and processes across landscapes. The increasing availability of medium-resolution satellite imagery (e.g., Landsat and Sentinel) and cloud-based platforms such as Google Earth Engine enables reproducible workflows for long-term monitoring (Gorelick et al., 2017; Wulder et al., 2022). Supervised classification approaches, particularly those using machine learning algorithms like Random Forest, have been widely adopted for land cover mapping due to their robustness and efficiency (Azzari & Lobell, 2017; Belgiu & Drăguţ, 2016). These methods are especially valuable in regions lacking systematic maps or when studying periods prior to their creation (Yang et al., 2017), and they often outperform coarse-scale products in small study areas (< 100 km^2^), where systematic maps can introduce substantial errors (Catalá-Mateo et al., 2008; Falťan et al., 2020). Yet, despite these advantages and their increasing use, few studies have compared remote sensing–based classifications with systematic maps in terms of accuracy and temporal consistency across the multiple spatial scales at which freshwater ecological processes operate.

This study addresses that gap by evaluating the reliability of systematic land cover maps for ecological assessment and testing whether Landsat-based supervised classification can provide robust indicators of land cover trajectories. We focus on five headwater catchments (< 70 km^2^) within Natura 2000 protected areas in northern Spain, representing ecologically sensitive landscapes where land cover strongly influences freshwater ecosystem condition. Using Google Earth Engine and Random Forest, we reconstructed land cover dynamics over four decades (1984–2023) and compared these results with CORINE, SIOSE, and the Spanish National Forest Inventory across three ecologically relevant spatial scales: catchment, riparian, and reach. Specifically, we aim to (i) quantify classification accuracy and temporal trends derived from supervised classification; (ii) assess discrepancies between systematic maps and remote sensing–based classifications in land cover proportions and trends; and (iii) examine how these discrepancies vary with spatial scale, with implications for interpreting landscape patterns and ecological processes. We hypothesised that (i) supervised classification would achieve higher accuracy than systematically produced maps across all periods; (ii) discrepancies between systematic maps and supervised classification would increase at finer spatial scales (riparian and reach), reflecting scale-dependent biases; and (iii) systematic maps would misrepresent temporal trends, potentially compromising assessments of landscape–ecosystem relationships.

## Materials and methods

### Study area

The study was conducted in five headwater catchments located in the northern Iberian Peninsula (Table S1), all fully or partially included within Natura 2000 protected areas (European Commission, 1992; 2009). These catchments range in size from 39 to 69 km^2^ and represent ecologically sensitive landscapes where land cover strongly influences freshwater ecosystem condition. Three catchments—Aizkorri, Aralar, and Artikutza—are situated in the temperate bioclimatic region, whereas Gorbea and Izki belong to the Mediterranean region, resulting in marked differences in dominant vegetation and land use patterns. The landscapes are semi-natural, characterised by a mosaic of natural forests, plantation forests, grasslands, and agricultural areas, interspersed with small urban settlements and water bodies.

Eight land cover categories were considered: (1) water, primarily reservoirs; (2) urban areas, including villages and small infrastructures; (3) bare soil, such as rocky peaks and quarries; (4) grassland, including meadows and hayfields; (5) agricultural and cereal croplands; (6) shrubland and low woody vegetation; (7) natural forest, largely deciduous broadleaf stands; and (8) plantation forest, dominated by *Pinus radiata* D. Don. These categories were selected because they represent key drivers of stream ecological integrity across multiple spatial scales. At the catchment scale, land cover influences hydrological processes and nutrient fluxes, thereby shaping biodiversity patterns (Mello et al., 2018; Weigel et al., 2023). At the riparian scale, defined here as a 30 m buffer along the entire stream network, land cover determines shading, organic matter input, and habitat quality, which are critical for maintaining stream ecological integrity (Sweeney & Newbold, 2014). At the reach scale, represented by a 100 m buffer around ten uniformly distributed points along each catchment’s river network, land cover captures local conditions that influence aquatic communities and ecosystem functioning (Leitão et al., 2018).

### Data sources

#### Satellite imagery

We used Landsat imagery from missions 4, 5, 7, and 8, covering the period 1984–2023. Data were obtained from the Level-2, Collection 2, Tier 1 dataset (EROS, 2020a, 2020b, 2020c), which provides atmospherically corrected surface reflectance products suitable for long-term time-series analysis. Landsat was selected because of its extensive historical archive, global coverage, and consistent acquisition since the 1980 s, making it particularly valuable for reconstructing multi-decadal land cover trajectories (Wulder et al., 2022). Specifically, Landsat 4–5 Thematic Mapper (TM) provided data from 1984 to 2012, Landsat 7 Enhanced Thematic Mapper Plus (ETM+) from 1999 onwards, and Landsat 8 Operational Land Imager/Thermal Infrared Sensor (OLI/TIRS) from 2013 onwards.

#### Systematic maps

Three systematically produced land cover datasets were used for comparison with supervised classification results. CORINE Land Cover (CLC), developed under the Copernicus Programme, provides harmonised land cover maps for Europe at a scale of 1:100,000 and a minimum mapping unit (MMU) of 25 ha, with five temporal versions available between 1990 and 2018 (Büttner et al., 2004). SIOSE (Sistema de Información de Ocupación del Suelo) is a Spanish national land occupation database that integrates multiple sources, including orthophotos and official cartography, with spatial resolution ranging from 1:25,000 to 1:1000–5000 depending on the region and year; five updates are available between 2004 and 2017 (Bosque González et al., 2005). In its last version, which provides data from 2017, SIOSE adopted the new high-resolution framework based on the integration of multiple official geospatial datasets, replacing the previous photointerpretation-based workflow (Spanish Geographical Institute; Instituto Geográfico Nacional, 2023). This transition also increased the effective spatial detail of the product (Table S2). The Spanish National Forest Inventory (NFI) combines digital photointerpretation with field sampling and provides detailed information on forest cover type, density, and land use; in the Basque Country, the NFI is produced at a finer scale (1:10,000) while maintaining compatibility with the national 1:25,000 scale (Álvarez-González et al., 2014) (Table S2).

#### Topographic data

Three static topographic variables—elevation, slope, and aspect—were derived from a LiDAR-based digital terrain model (DTM) with a spatial resolution of 5 m, obtained from the Spanish National Geographic Institute (IGN.es). These variables provide essential context for land cover classification, as topography influences vegetation distribution and spectral responses.

### Image pre-processing and composite generation

Landsat scenes covering the study area with ≤ 50% cloud cover were selected. Cloud and shadow masking was performed using the QA_PIXEL band, which identifies low-quality pixels based on cloud, cloud shadow, and other atmospheric artefacts. Surface reflectance values were rescaled to the [0,1] range using the scaling factors recommended by USGS. Images were grouped into eight approximately 5-year periods spanning from 1984 to 2023, referred to by the central year: 1987, 1992, 1997, 2002, 2007, 2012, 2017, and 2021. Each period was further divided into four seasonal composites (January–March, April–June, July–September, and October–December) to capture phenological variability, which enhances the discrimination of land cover classes such as deciduous and evergreen vegetation. Each seasonal composite included six optical reflectance bands common to all Landsat sensors (Blue, Green, Red, Near Infrared, Shortwave Infrared 1, and Shortwave Infrared 2) and ten spectral indices designed to improve separability between classes (Table S3). These indices included NDVI (Rouse et al., 1974), SAVI (Huete, 1988), NDWI (McFeeters, 1996), NDBI (Zha et al., 2003), GCVI (Gitelson et al., 2003), BSI (Nguyen et al., 2021), SIPI (Peñuelas & Filella, 1998), and three additional normalised differences between Green/SWIR, SWIR1/SWIR2, and NIR/SWIR2 bands. Together with the three topographic variables, this approach yielded 67 features per pixel, providing a rich dataset for classification (Table S4).

### Reference data and temporal consistency

A total of 1089 reference points were manually selected using a stratified random sampling method from a cloud-free summer 2016 Landsat image, representing the eight land cover categories. These samples were identified through visual interpretation of high-resolution orthophotography (0.25 m) from the Basque Government and Government of Navarre Spatial Data Warehouses (GeoEuskadi & IDENA). Seventy percent of the samples were used for model training and 30% for validation. To apply these samples across the full study period, we used a spectral angle distance (SAD) approach to quantify spectral similarity between each sample and its 2016 reference value (Huang et al., 2020). Samples with SAD values greater than 0.25 were excluded from training or validation in each year because they were considered spectrally dissimilar, potentially indicating land cover change or acquisition-related anomalies (Feng et al., 2024). This filtering threshold ensured temporal consistency in the reference dataset and strengthened the robustness of the resulting classifications.

### Supervised classification

Classification was performed using the Random Forest algorithm (Breiman, 2001), implemented in Google Earth Engine (Gorelick et al., 2017). Random Forest was selected for its robustness, efficiency, and suitability for high-dimensional datasets, as well as its proven performance in remote sensing applications (Belgiu & Drăguţ, 2016). The model was configured with 200 trees, while other parameters were kept at defaults. Because the objective was temporal consistency of land cover trajectories rather than maximising predictive accuracy for a single period, single classification iteration was run, with no cross-validation applied.

### Accuracy assessment

Classification accuracy was evaluated using three metrics: overall accuracy (OA, Eq. 1), producer’s accuracy (PA, Eq. 2), and user’s accuracy (UA, Eq. 3) (Stehman & Foody, 2019). OA represents the proportion of correctly classified validation points, while PA and UA provide class-specific accuracy information, corresponding to omission and commission errors, respectively. The metrics were calculated as follows:

1 $$OA=\frac{{N}_{correct}}{{N}_{total}}\times 100$$

2 $$PA=\frac{{N}_{predicted class=reference class}}{{N}_{reference class}}\times 100$$

3 $$UA=\frac{{N}_{predicted class=reference class}}{{N}_{predicted class}}\times 100$$

An OA ≥ 85% was considered reliable following the threshold proposed by Anderson et al. (1976). Accuracy was assessed for each temporal period and averaged across all periods to evaluate consistency over time.

### Comparison with systematic maps

Land cover categories from CORINE, SIOSE, and NFI were reclassified to match the supervised classification legend (Tables S5–S7). We compared land cover area percentages and temporal trends across the three spatial scales (catchment, riparian, and reach) using linear models. For both supervised classification and systematic maps, linear models were fitted to describe temporal changes for each land cover class within each catchment and scale. Agreement between supervised classification and systematic maps was tested against a restricted model with slope = 1 and intercept = 0, representing perfect equivalence, using *F*-tests. This approach allowed us to quantify discrepancies in both absolute area estimates and trend slopes, highlighting potential biases introduced by systematic maps.

### Data and code availability

To ensure transparency and reproducibility, all Google Earth Engine scripts and R codes used for classification and analysis are publicly available. The GEE script can be accessed at https://code.earthengine.google.com/dae0da193274d6a20f4da1efa2e32f74, and additional resources, including training point sets and validation data, are available in a public GitHub repository (https://github.com/inakifdzlarrea/land_cover_assessment).

## Results

### Classification accuracy

The supervised classification achieved high accuracy across all eight temporal periods (1984–2023). Overall accuracy (OA) ranged from 80.7% in the earliest period (1984–1989) to 92.1% in 2005–2009, with intermediate values of 82.8% (1990–1994), 85.3% (1995–1999), 90.0% (2000–2004), 90.3% (2010–2014), 91.6% (2015–2018), and 89.6% (2019–2023) (Table 1). The mean OA across all periods was 87.8%. The number of Landsat scenes increased from 136 in 1984–1989 to 287 in 2019–2023, while the number of reference points after SAD filtering ranged between 930 and 1038 per period (Table S8). Class-specific accuracy metrics showed consistent performance for most categories. Water achieved 100% producer’s accuracy (PA) and user’s accuracy (UA) across all periods. Urban areas, natural forest, and agriculture also performed well, each exceeding 91% in PA and UA on average. Bare soil reached 85.3% PA and 93.5% UA, while plantation forest achieved 88.4% PA and 86.6% UA. Grassland and shrubland exhibited lower accuracies, with grassland averaging 81.4% PA and 83.7% UA and shrubland 78.2% PA and 74.9% UA (Table 2). Temporal variability was greatest for shrubland, with PA differences of up to 10.4% across periods, whereas water and urban classes remained stable (< 5% variation; Table S9). Misclassifications primarily involved confusion between shrubland and grassland or natural forest (Table S10).

**Table 1:** Overall accuracy (%) of supervised classification for each temporal period (1984–2023) and the mean accuracy across all periods

Period	Overall accuracy (%)
1984–1990	80.7
1990–1995	82.8
1995–2000	85.3
2000–2005	90.0
2005–2010	92.1
2010–2015	90.3
2015–2019	91.6
2019–2023	89.6
**All periods (1984**–**2023)**	**87.8**

**Table 2:** Mean producer’s accuracy (PA) and user’s accuracy (UA) for each land cover class across all periods, including average temporal variation in PA and UA

Land use	Mean PA	Mean UA	Average PA difference	Average UA difference
Agriculture	92.6	91.8	3.4	5.1
Bare soil	85.3	93.5	9.8	3.7
Grassland	81.4	83.7	6.5	9.0
Natural forest	91.6	92.6	2.7	3.0
Plantation forest	88.4	86.6	3.2	5.1
Shrubland	78.2	74.9	10.4	6.9
Urban	92.4	92.9	5.2	4.2
Water	100.0	100.0	0.0	0.0

#### Land cover composition

**Fig. 1:**
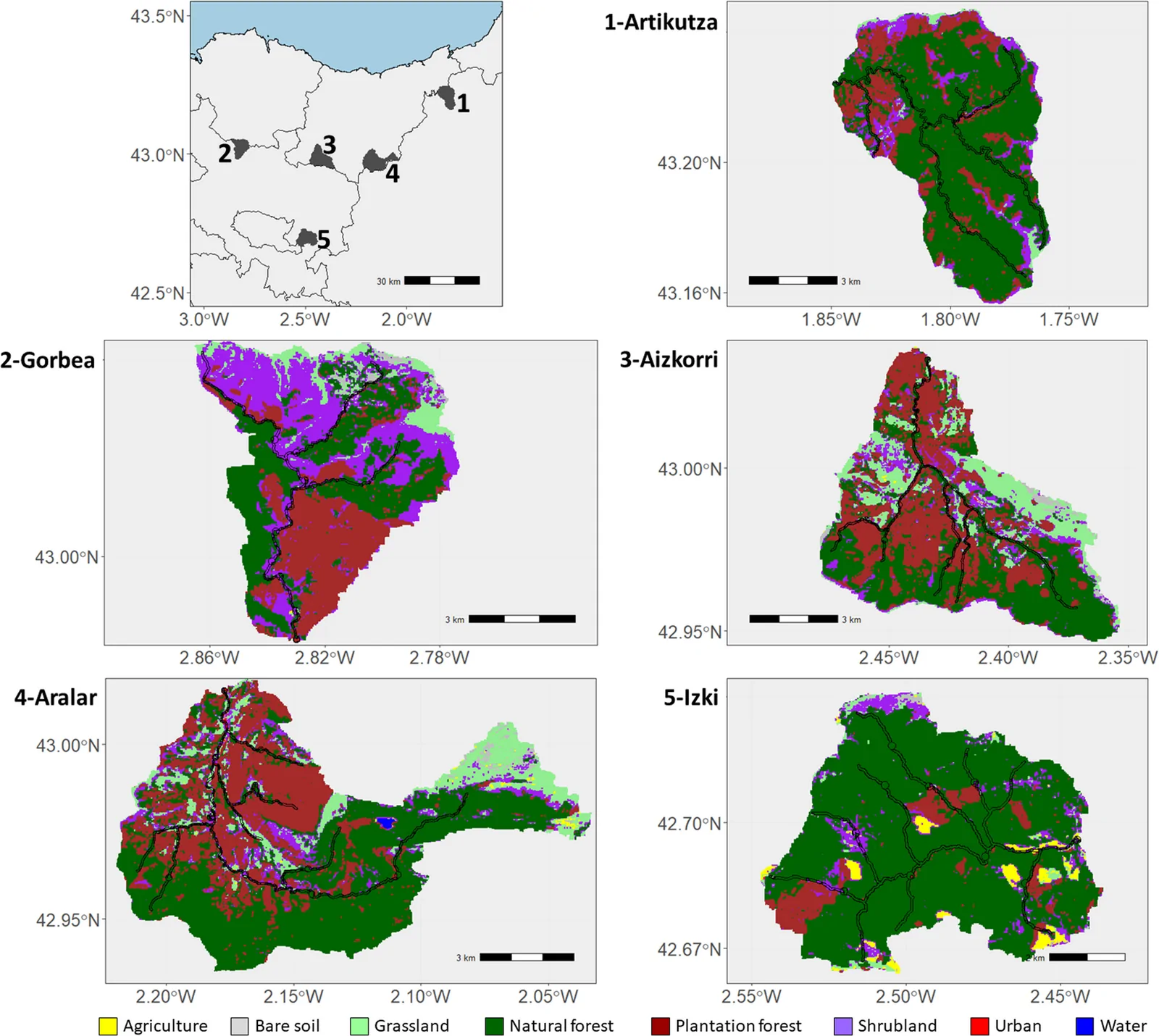
Location of the five catchment areas and the land cover estimated by means of SC in each one of them for the last period in this study (2019–2023)

**Fig. 2:**
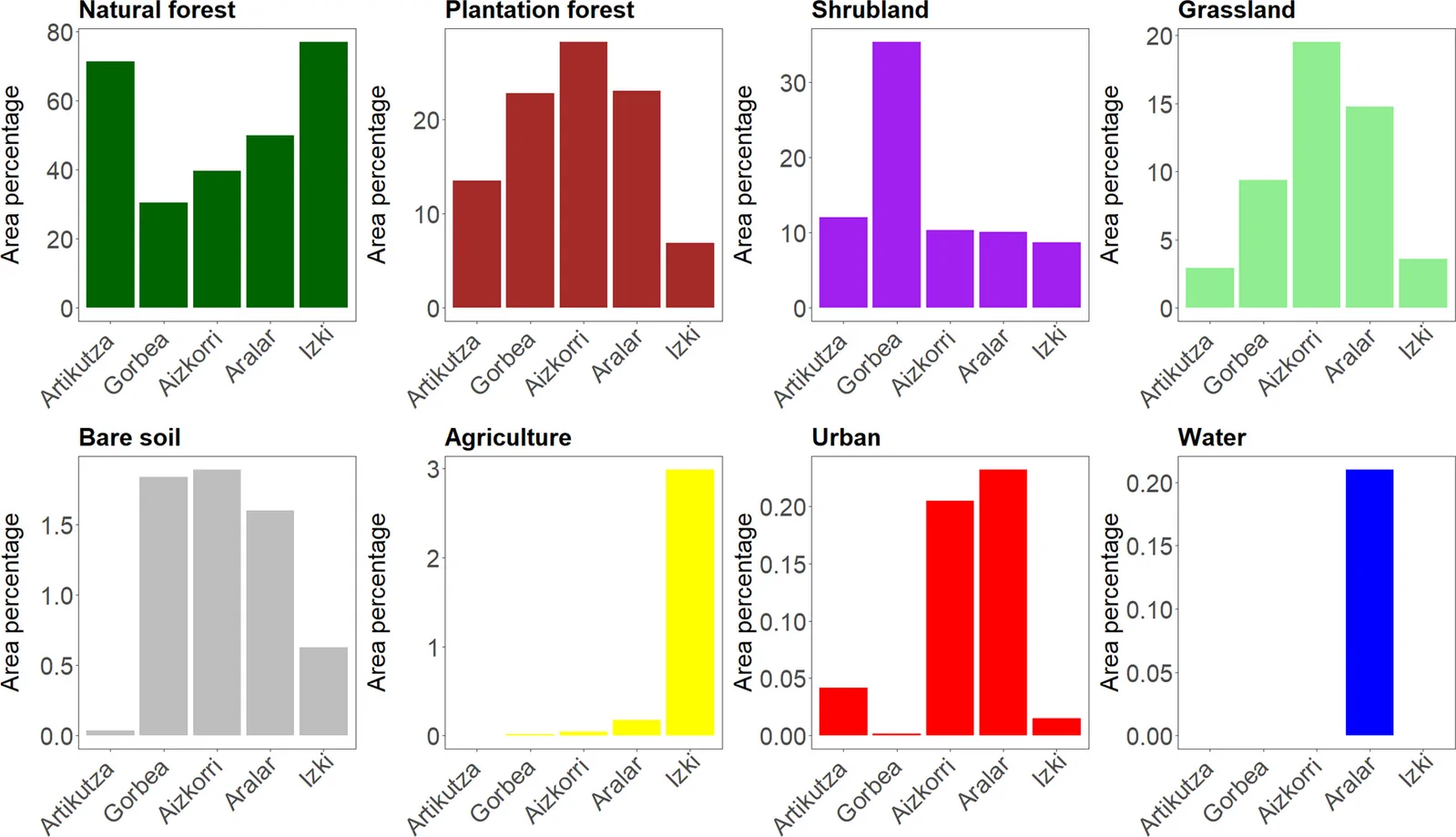
Estimated land cover area percentage (%) at the catchment scale in the most recent period (2019–2023) for each land cover category. Land cover categories ordered from the most to the least abundant. Colours match the ones shown in Fig. 1

For the most recent period (2019–2023), natural forest was the dominant land cover across all catchments and scales, forming the core matrix of the landscape (Fig. 1). At the catchment scale, natural forest accounted for 52.9% of the total area, followed by plantation forest (14.8%), shrubland (12.3%), grassland (10.7%), agriculture (5.6%), urban areas (2.1%), bare soil (1.2%), and water (0.4%) (Fig. 2; Table S11). Riparian zones exhibited higher proportions of natural forest (65.0%) highlighting the importance of linear corridors for habitat connectivity and stream ecosystem integrity, whereas they showed lower proportions of grassland (8.4%) and agriculture (3.2%) compared to catchments (Table S12). At the reach scale, natural forest represented 52.2%, shrubland 14.1%, plantation forest 13.6%, and grassland 11.2%, with other classes contributing less than 5% (Table S13). Catchment-level patterns varied among sites. Aizkorri and Aralar showed substantial shares of plantation forest, while Gorbea exhibited extensive shrubland. Grassland was most prominent in Aralar and Izki, whereas agriculture was concentrated in Izki. Urban cover was limited overall but highest in Aizkorri and Aralar. Water and bare soil were residual across all catchments.

### Temporal trends

**Fig. 3:**
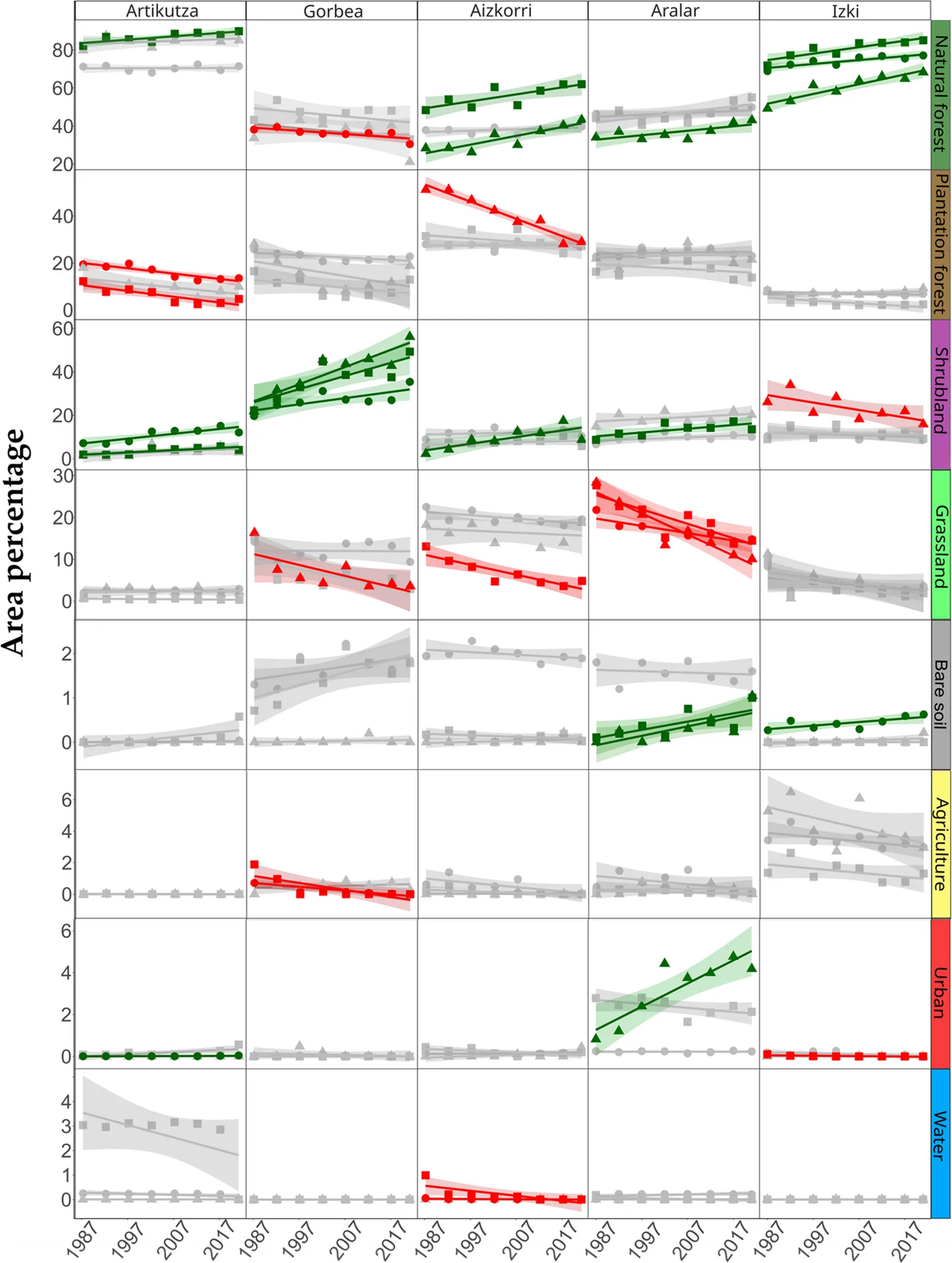
Land cover area percentage estimated by SC at 5 protected areas for each land cover at 3 scales—catchment (circle), riparian (square), and reach (triangle). Linear model representing the temporal trend of each land cover at each scale and protected area: green lines are significant positive trends, red lines are significant negative trends, and grey lines are not significant trends

Analysis of temporal dynamics revealed significant changes in land cover composition over the 40-year period (Fig. 3). Among the 120 fitted linear models (five catchments × eight land cover classes × three scales), 19 exhibited significant positive trends and 15 significant negative trends (Table S14). Natural forest and shrubland accounted for the largest number of positive trends (six each), indicating progressive expansion. Bare soil increased in Aralar (reach and riparian) and Izki (catchment), while urban areas expanded at the reach scale in Aizkorri and Aralar. Grassland showed the highest number of decreasing trends (five), and plantation forest declined in Aizkorri and Artikutza. In Gorbea, agriculture, natural forest, and grassland also exhibited decreasing trends. Slopes of fitted models varied by class and scale, with natural forest increases ranging from 0.3% to 1.2% per decade and grassland decreases from − 0.4% to − 1.0% per decade. As general overview, trend magnitudes varied by spatial scale, illustrating scale-dependent changes in patch composition and configuration.

#### Comparison with systematic maps

**Fig. 4:**
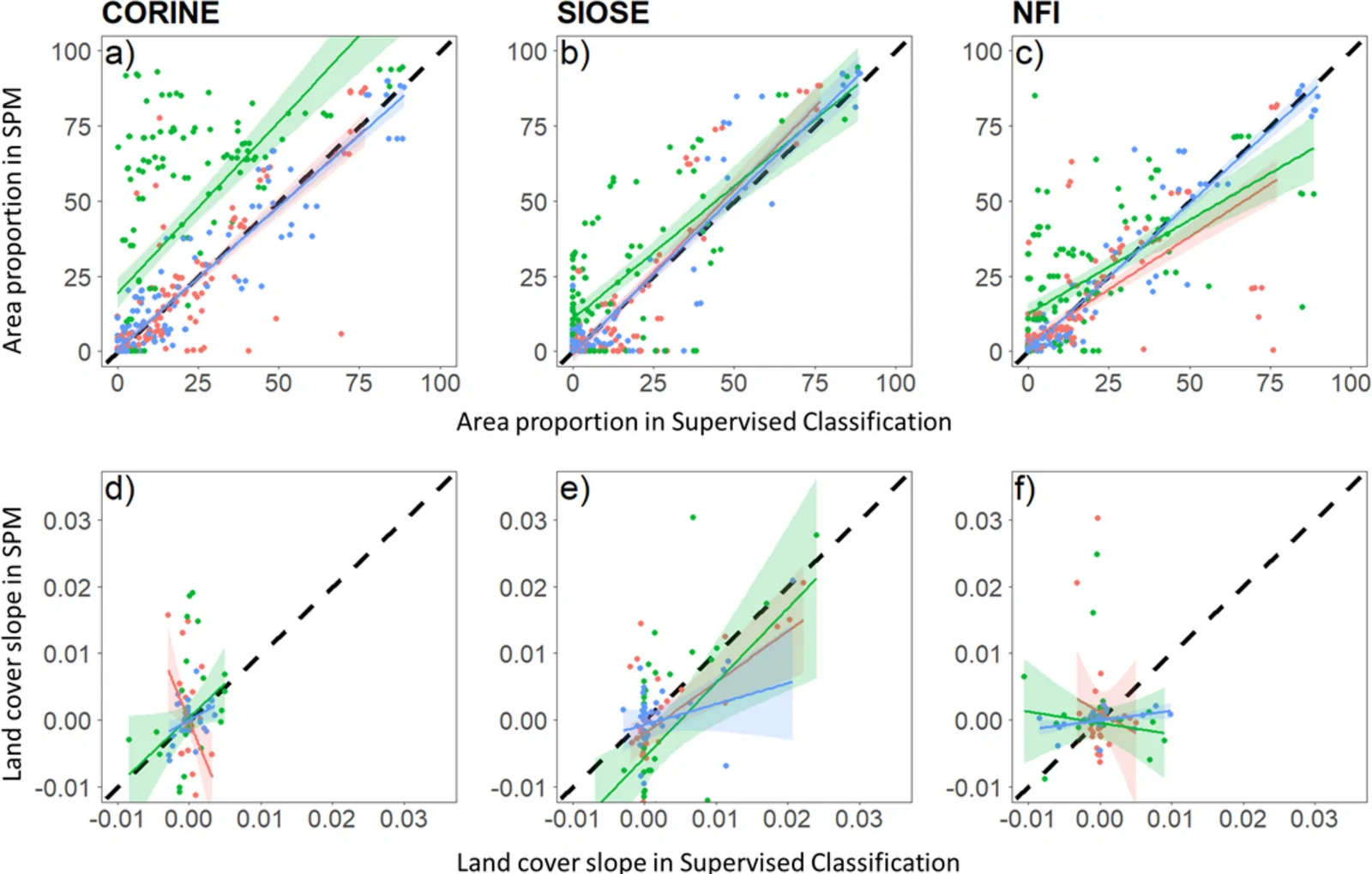
Land cover area percentage (**a**, **b**, **c**) and the slope of the linear model assessing temporal trend (**d**, **e**, **f**) values obtained in SC compared to CORINE (**a**, **d**), SIOSE (**b**, **e**), and NFI (**c**, **f**). Linear models show the fitness between the results obtained by systematically produced maps (SPM) and supervised classification at three spatial scales: catchment (red), riparian (blue), and reach (green)

Accuracy comparisons between SC and systematically produced maps revealed marked differences (Table 3). Across shared periods (2005–2018), SC achieved a mean OA of 91.3%, outperforming CORINE (71.0%) and SIOSE (64.7%), and matching NFI (91.5%). CORINE accuracy ranged from 62.1% in 1990–1994 to 73.1% in 2015–2018, while SIOSE improved from 54.0% in 2005–2009 to 88.3% in 2015–2018. NFI maintained consistently high accuracy (> 90%) but is limited to forest categories. Because of differing classification accuracies among products, the use of different maps leads to diverging land cover estimates (Figure S1). Discrepancies in land cover area estimates were most pronounced at finer scales (reach), where systematic maps diverged significantly from SC (Fig. 4a–c). CORINE and SIOSE underestimated narrow riparian features and fragmented habitats due to coarse MMUs and semantic inconsistencies. At the catchment scale, deviations were smaller but evident for shrubland and grassland. Riparian zones showed intermediate discrepancies. Linear models comparing temporal trend slopes revealed further mismatches. CORINE exhibited the largest divergence; with fitted slopes differing significantly from the expected 1:1 relationship at all scales (Fig. 4d). SIOSE showed apparent visual agreement but failed statistical equivalence tests, while NFI discrepancies were most noticeable at riparian and reach scales (Fig. 4f; Table S15). *F*-tests confirmed significant deviations for most comparisons (*p* < 0.05). These discrepancies indicate that assessments based on systematic maps would yield substantially different conclusions regarding land cover dynamics at management-relevant scales.

**Table 3:** Overall accuracy (%) of supervised classification (SC) and systematically produced maps (CORINE, SIOSE, NFI) by period. Mean accuracy and average accuracy difference are calculated for periods where all maps are available (2005–2009, 2010–2014, 2015–2018)

Period	SC	CORINE	SIOSE	NFI
1984–1989	80.7			
1990–1994	82.8	62.1		
1995–1999	85.3			
2000–2004	90.0	65.8		
2005–2009*	92.1	66.1	54.0	92.2
2010–2014*	90.3	73.9	51.8	90.3
2015–2018*	91.6	73.1	88.3	92.1
2019–2023	89.6			92.3
Mean accuracy*	91.3	71.0	64.7	91.5
Average accuracy difference*	0.7	3.3	15.7	0.8

## Discussion

Systematically produced land cover maps are widely used by researchers and managers because they provide accessible, harmonised datasets for large regions (Pandey et al., 2021; Wang et al., 2023). A Web of Science search conducted on 2 April 2025 yielded 944 documents related to ecology and biodiversity conservation that used CORINE, SIOSE, or NFI, underscoring the potential impact of inaccuracies in these maps on research outcomes and management decisions. However, the implications of using coarse-scale maps vary with context. For example, Seoane et al., (2004) found that modelling bird species distributions using coarse-scale data introduced only minor biases, whereas Hernando et al. (2017) reported significant effects of map inaccuracies on forest fragmentation and connectivity for habitat conservation status assessments. Similarly, Di Sabatino et al., (2013) demonstrated that CORINE data substantially underestimated freshwater ecosystems and their associated services. These findings emphasise the need to evaluate whether systematically produced maps adequately represent landscape structure at the spatial scales relevant to ecological processes in river ecosystems.

Our results demonstrate that supervised classification (SC) provides a more reliable representation of land cover patterns across multiple spatial scales. SC achieved consistently higher accuracy than systematic maps across all periods, with a mean overall accuracy of 87.8%, above the 85% reliability threshold proposed by Anderson et al. (1976). This performance aligns with other Landsat-based SC studies (Li et al., 2020; Phan et al., 2020; Xie et al., 2019) and reflects the robustness of Random Forest algorithms for high-dimensional datasets (Azzari & Lobell, 2017; Belgiu & Drăguţ, 2016). In contrast, CORINE and the early SIOSE versions exhibited substantially lower accuracies (averaging 71.0% and 52.9%, respectively). A substantial improvement was observed for SIOSE in the 2015–2018 period (88.3% overall accuracy), which coincides with the introduction of SIOSE High Resolution framework (Spanish National Geographical Institute; Instituto Geográfico Nacional, 2023). Meanwhile, NFI performed comparably to SC only for forest categories. These results confirm that systematic maps, especially when multiple data sources are combined, are valuable for broad-scale assessments, but they are less suitable for analyses requiring accurate representation of fine-grained landscape mosaics or linear features, such as riparian corridors (Catalá-Mateo et al., 2008; García Álvarez & Camacho Olmedo, 2023).

Discrepancies between systematic maps and SC were scale-dependent but context-specific. As expected, the largest differences occurred at the reach scale, where narrow spatial extents amplify classification errors (Tormos et al., 2011). However, discrepancies at the catchment scale were sometimes greater than those observed in riparian zones, likely reflecting the relative stability of riparian vegetation (Bhatti et al., 2015), which can be associated with management measures implemented under the EU Water Framework Directive (European Commission, 2000). This suggests that sensitivity to mapping bias arises not only from spatial scale but also from landscape dynamics and heterogeneity, reinforcing the need for scale-aware land cover representations when assessing freshwater ecosystems embedded within heterogeneous landscapes.

Systematic maps also showed limited temporal consistency. Compared with SC, they failed equivalence tests for most spatial scales, indicating that long-term trends were frequently misrepresented. CORINE exhibited the largest divergence, with trend slopes differing significantly from the expected 1:1 relationship, while SIOSE showed apparent agreement that was not supported statistically. Such inconsistencies can bias interpretations of landscape change, for example by underestimating forest expansion or grassland decline, and may lead to contrasting conclusions about connectivity, matrix composition, or habitat availability over time (Rodríguez-Rodríguez & Martínez-Vega, 2018; Vogiatzakis et al., 2015).

Beyond accuracy, these biases have important implications for landscape–ecosystem relationships. Land cover strongly influences freshwater ecosystem condition through hydrological regulation, nutrient fluxes, and habitat quality (Leitão et al., 2018; Mello et al., 2018; Sweeney & Newbold, 2014). Misrepresentation of riparian corridors or fragmented habitats can distort assessments of connectivity, edge effects, and functional integrity, particularly in heterogeneous headwater landscapes where ecological processes operate at fine spatial scales (Vidal-Llamas et al., 2025). For instance, inaccurate representation of riparian vegetation may lead to incorrect assessments of shading and organic matter inputs, which are critical for stream ecological functioning. Similarly, errors in grassland or shrubland mapping can alter connectivity metrics and biodiversity models, cascading into flawed conservation strategies. These risks are particularly relevant given global biodiversity targets and the need for robust indicators under IPBES frameworks (IPBES, 2019).

Methodologically, this study provides a reproducible workflow for generating accurate land cover indicators for landscape ecological analyses and freshwater ecosystem assessment using freely available satellite imagery and cloud-based platforms. The integration of Random Forest within Google Earth Engine enables transparent, scalable analyses (Gorelick et al., 2017), while Landsat long-term archive supports retrospective assessments essential for detecting legacy effects (Bürgi et al., 2017; Janssen et al., 2018; Martin et al., 2017).

### Limitations of the study

Despite its advantages, the SC approach presents limitations. Its performance declined for spectrally similar classes such as shrubland and grassland, reflecting known challenges associated with medium-resolution imagery (Gao et al., 2020; Zhao et al., 2023). In addition, this study was conducted at a fine spatial scale, as its primary aim was to highlight the limitations of systematically produced maps at this scale, which are typically designed for coarser resolutions. Therefore, it cannot be assumed that SC would consistently outperform these products at broader spatial scales. Another limitation relates to image availability: the SC routine relies on Landsat data accessible through Google Earth Engine, restricting temporal analyses to the period from 1984 onward and preventing earlier reconstructions. Furthermore, the classification scheme was limited to a specific set of land cover categories relevant to ecology. This may constrain its direct applicability to other contexts, requiring adjustments to category definitions according to the study’s objectives. Future improvements could include the integration of higher-resolution imagery (e.g. Sentinel-2) and ancillary datasets such as LiDAR to enhance classification accuracy and scalability.

## Conclusions

This study demonstrates that reliance on systematically produced land cover maps can introduce scale-dependent and context-specific biases that affect the interpretation of riparian landscape patterns and their ecological implications, particularly in riparian systems. By comparing CORINE, SIOSE, and NFI with a Landsat-based supervised classification (SC), we demonstrate that SC provides consistently higher accuracy, that discrepancies with systematic products intensify at finer spatial scales, and that temporal trends derived from coarse-scale maps can diverge substantially from those obtained using remote sensing–based approaches. These findings highlight the need for caution when using systematic land cover products in landscape ecological studies that seek to link spatial pattern with ecological processes at local to regional scales. Integrating SC-based, scale-aware land cover representations into monitoring and assessment frameworks offers a practical pathway to improving the reliability of landscape indicators and their relevance for both ecological inference and policy implementation. Continued methodological development—particularly to improve discrimination among spectrally similar classes and to incorporate higher-resolution data—will further enhance the capacity of SC workflows to support robust, cross-scale landscape ecological analyses.

## Supplementary Information

Below is the link to the electronic supplementary material.

ESM 1 DOCX (1.58 MB)

## Data Availability

The Google Earth Engine code developed for our Supervised Classification routine can be found in the following link: (https:/code.earthengine.google.com/dae0da193274d6a20f4da1efa2e32f74). Files containing information about the training point set, land cover results and R-codes have been uploaded to a public GitHub repository: (https:/github.com/inakifdzlarrea/land_cover_assessment.git). For the replicability of the search on Web of Science conducted in 2nd April 2025 we shared the link: (https:/www.webofscience.com/wos/woscc/summary/a099406f-36ef-4697-ba65-96a1f0058c86-0150e14003/relevance/1).
