# Supplementary material for: Scale-dependent biases in systematic land cover maps undermine freshwater ecological assessment
Source: Environ Monit Assess. 2026 Sep 14;198(10):1071. doi: 10.1007/s10661-026-15882-1 (PMC13574915; doi:10.1007/s10661-026-15882-1)
Supplement: Supplementary file 1 [file 10661_2026_15882_MOESM1_ESM.docx]

**Supplementary material**

**Scale-Dependent Biases in Systematic Land Cover Maps Undermine Freshwater Ecological Assessment**

**Landscape Ecology**

Authors: Iñaki Fernández de Larrea^1*^, Jon González-Ibarzabal^2^, Aitor Bastarrika^2,3^ & Aitor Larrañaga^1^.

^1^Department of Plant Biology and Ecology, Faculty of Science and Technology, University of the Basque Country (UPV/ EHU), Bº Sarriena s/n, 48940 Leioa, Spain.

^2^Built Heritage Research Group, University of the Basque Country UPV/EHU, C/Nieves Cano, 12, 01006 Vitoria-Gasteiz, Spain.

^3^Department of Mining and Metallurgical Engineering and Materials Science, School of Engineering of Vitoria-Gasteiz, University of the Basque Country (UPV/ EHU), C/Nieves Cano, 12, 01006 Vitoria-Gasteiz, Spain.

*****Corresponding author: Iñaki Fernández de Larrea; inaki.fernandezdelarrea@ehu.eus

**Table S1** Geographic coordinates and draining catchment area (km^2^) of the outflow point of the five catchments selected for this study. Code of the Natura 2000 site and the bioclimatic region for each catchment is indicated.

| **Catchment** | **Long.** | **Lat.** | **Area (km^2^)** | **Natura 2000 site code** | **Bioclimatic region** |
| --- | --- | --- | --- | --- | --- |
| Aizkorri | 2°25’48’’W | 43°13’64’’N | 53.61 | ES2120002 | Temperate |
| Aralar | 2°10’48’’W | 43°00’58’’N | 69.52 | ES2120011 | Temperate |
| Artikutza | 1°51’00’’W | 43°01’59’’N | 47.09 | ES2200010 | Temperate |
| Gorbea | 2°49’48’’W | 42°58’41’’N | 39.17 | ES2110009 | Mediterranean |
| Izki | 2°26’24’’N | 42°41’46’’N | 43.60 | ES2110019 | Mediterranean |

**Table S2** Key characteristics of the systematically produced land cover datasets used for comparison in this study. The table summarizes spatial resolution or scale, mapping periods, number of available maps, producing institutions, minimum mapping units (MMU), classification approaches, and thematic content (legend/class semantics) for CORINE, SIOSE, and the Spanish National Forest Inventory (NFI).

| Product Name | Spatial Resolution / Scale | Mapping Period | Nº of Maps | Developer / Authority | MMU | Classification Logic | Legend / Class Semantics |
| --- | --- | --- | --- | --- | --- | --- | --- |
| CORINE Land Cover | 25 ha / 100 m; 1:100,000 | 1990–2018 | 5 | Copernicus Program / CNIG (Spain) | 25 ha (areal), 100 m (linear) | Image interpretation of satellite imagery | 3-level hierarchical classification (44 classes) |
| SIOSE | 1:25,000 (nominal) ; 1:1,000–5,000 (2017) | 2004–2017 | 5 | CNIG and Regional Governments | 1–5 ha depending on source | Multi-source integration (orthophotos, cartography, satellite) | Rich attribute table; complex, harmonized at national scale |
| National Forest Inventory (NFI) | 1:25000 (nominal);1:10,000 – (Basque Country) | 2005–2024 | 8* | Spanish Government (Basque Gov. for 2018–present) | Not MMU-based; plot and photo-interpretation | Field plots + photo-interpretation | Forest-focused; class detail based on structure and composition |

*Since 2018, the National Forest Inventory has been updated every year in the Basque Country, but there are only 2 available maps until 2017.

**Table S3** Equations of the 10 spectral indices derived from Landsat reflectance bands and used in the supervised classification. These indices are commonly applied to enhance separability between vegetation, water, soil, and urban surfaces.

| **Index** | **Equation** |
| --- | --- |
| NDVI | (NIR - RED) / (NIR + RED) |
| GCVI | (NIR / GREEN) - 1 |
| BSI | ((SWIR1 + RED) - (NIR + BLUE)) / ((SWIR1 + RED) + (NIR + BLUE)) |
| NDBI | (SWIR1 - NIR) / (SWIR1 + NIR) |
| SAVI | ((NIR - RED) / (NIR + RED + L)) * (1 + L); L = 0.5 |
| NDWI | (GREEN - NIR) / (GREEN + NIR) |
| SIPI | (NIR - BLUE) / (NIR - RED) |
| GREEN-SWIR1 | (GREEN - SWIR1) / (GREEN + SWIR1) |
| SWIR1-SWIR2 | (SWIR1 - SWIR2) / (SWIR1 + SWIR2) |
| NIR-SWIR2 | (NIR - SWIR2) / (NIR + SWIR2) |

**Table S4** Description of the 67 variables used in the Supervised Classification. For each of the four seasons (January–March, April–June, July–September, and October–December), six surface reflectance bands and ten spectral indices were calculated from Landsat imagery. Additionally, three topographic variables (elevation, slope, and aspect) derived from a 5 m-resolution digital terrain model (DTM) were included, resulting in a total of 67 input features per pixel.

| **Band No.** | **Variable Name** | **Type** |
| --- | --- | --- |
| 1 | Winter (Jan-Mar) - Blue | Reflectance band |
| 2 | Winter (Jan-Mar) - Green | Reflectance band |
| 3 | Winter (Jan-Mar) - Red | Reflectance band |
| 4 | Winter (Jan-Mar) - NIR | Reflectance band |
| 5 | Winter (Jan-Mar) - SWIR1 | Reflectance band |
| 6 | Winter (Jan-Mar) - SWIR2 | Reflectance band |
| 7 | Winter (Jan-Mar) - NDVI | Spectral index |
| 8 | Winter (Jan-Mar) - GCVI | Spectral index |
| 9 | Winter (Jan-Mar) - BSI | Spectral index |
| 10 | Winter (Jan-Mar) - NDBI | Spectral index |
| 11 | Winter (Jan-Mar) - SAVI | Spectral index |
| 12 | Winter (Jan-Mar) - NDWI | Spectral index |
| 13 | Winter (Jan-Mar) - SIPI | Spectral index |
| 14 | Winter (Jan-Mar) - GREEN–SWIR1 | Spectral index |
| 15 | Winter (Jan-Mar) - SWIR1–SWIR2 | Spectral index |
| 16 | Winter (Jan-Mar) - NIR–SWIR2 | Spectral index |
| 17 | Spring (Apr-Jun) - Blue | Reflectance band |
| 18 | Spring (Apr-Jun) - Green | Reflectance band |
| 19 | Spring (Apr-Jun) - Red | Reflectance band |
| 20 | Spring (Apr-Jun) - NIR | Reflectance band |
| 21 | Spring (Apr-Jun) - SWIR1 | Reflectance band |
| 22 | Spring (Apr-Jun) - SWIR2 | Reflectance band |
| 23 | Spring (Apr-Jun) - NDVI | Spectral index |
| 24 | Spring (Apr-Jun) - GCVI | Spectral index |
| 25 | Spring (Apr-Jun) - BSI | Spectral index |
| 26 | Spring (Apr-Jun) - NDBI | Spectral index |
| 27 | Spring (Apr-Jun) - SAVI | Spectral index |
| 28 | Spring (Apr-Jun) - NDWI | Spectral index |
| 29 | Spring (Apr-Jun) - SIPI | Spectral index |
| 30 | Spring (Apr-Jun) - GREEN–SWIR1 | Spectral index |
| 31 | Spring (Apr-Jun) - SWIR1–SWIR2 | Spectral index |
| 32 | Spring (Apr-Jun) - NIR–SWIR2 | Spectral index |
| 33 | Summer (Jul-Sep) - Blue | Reflectance band |
| 34 | Summer (Jul-Sep) - Green | Reflectance band |
| 35 | Summer (Jul-Sep) - Red | Reflectance band |
| 36 | Summer (Jul-Sep) - NIR | Reflectance band |
| 37 | Summer (Jul-Sep) - SWIR1 | Reflectance band |
| 38 | Summer (Jul-Sep) - SWIR2 | Reflectance band |
| 39 | Summer (Jul-Sep) - NDVI | Spectral index |
| 40 | Summer (Jul-Sep) - GCVI | Spectral index |
| 41 | Summer (Jul-Sep) - BSI | Spectral index |
| 42 | Summer (Jul-Sep) - NDBI | Spectral index |
| 43 | Summer (Jul-Sep) - SAVI | Spectral index |
| 44 | Summer (Jul-Sep) - NDWI | Spectral index |
| 45 | Summer (Jul-Sep) - SIPI | Spectral index |
| 46 | Summer (Jul-Sep) - GREEN–SWIR1 | Spectral index |
| 47 | Summer (Jul-Sep) - SWIR1–SWIR2 | Spectral index |
| 48 | Summer (Jul-Sep) - NIR–SWIR2 | Spectral index |
| 49 | Autumn (Oct-Dec) - Blue | Reflectance band |
| 50 | Autumn (Oct-Dec) - Green | Reflectance band |
| 51 | Autumn (Oct-Dec) - Red | Reflectance band |
| 52 | Autumn (Oct-Dec) - NIR | Reflectance band |
| 53 | Autumn (Oct-Dec) - SWIR1 | Reflectance band |
| 54 | Autumn (Oct-Dec) - SWIR2 | Reflectance band |
| 55 | Autumn (Oct-Dec) - NDVI | Spectral index |
| 56 | Autumn (Oct-Dec) - GCVI | Spectral index |
| 57 | Autumn (Oct-Dec) - BSI | Spectral index |
| 58 | Autumn (Oct-Dec) - NDBI | Spectral index |
| 59 | Autumn (Oct-Dec) - SAVI | Spectral index |
| 60 | Autumn (Oct-Dec) - NDWI | Spectral index |
| 61 | Autumn (Oct-Dec) - SIPI | Spectral index |
| 62 | Autumn (Oct-Dec) - GREEN–SWIR1 | Spectral index |
| 63 | Autumn (Oct-Dec) - SWIR1–SWIR2 | Spectral index |
| 64 | Autumn (Oct-Dec) - NIR–SWIR2 | Spectral index |
| 65 | Elevation | Topographic variable |
| 66 | Slope | Topographic variable |
| 67 | Aspect | Topographic variable |

**Table S5** Land cover category homogenization of CORINE to the categories defined on the Supervised Classification (SC). In brackets the name of the field used in each CORINE map to define land cover.

| **SC Category** | **CORINE Category 1990 (CODE_90)** | **CORINE Category 2000 (CODE_00)** | **CORINE Category 2006 (CODE_06)** | **CORINE Category 2012 (CODE_12)** | **CORINE Category 2018 (CODE_18)** |
| --- | --- | --- | --- | --- | --- |
| Urban | 111; 112; 121; 122; 123; 124; 131; | 111; 112; 121; 122; 123; 124; 131; | 111; 112; 121; 122; 123; 124; 131; | 111; 112; 121; 122; 123; 124; 131; | 111; 112; 121; 122; 123; 124; 131; |
|  | 132; 133; 141; 142 | 132; 133; 141; 142 | 132; 133; 141; 142 | 132; 133; 141; 142 | 132; 133; 141; 142 |
| Agriculture | 211; 212; 213; 221; 222; 223; 241; | 211; 212; 213; 221; 222; 223; 241; | 211; 212; 213; 221; 222; 223; 241; | 211; 212; 213; 221; 222; 223; 241; | 211; 212; 213; 221; 222; 223; 241; |
|  | 242; 243; 244 | 242; 243; 244 | 242; 243; 244 | 242; 243; 244 | 242; 243; 244 |
| Natural forest | 311; 313 | 311; 313 | 311; 313 | 311; 313 | 311; 313 |
| Plantation forest | 312 | 312 | 312 | 312 | 312 |
| Grassland | 231; 321; 412 | 231; 321; 412 | 231; 321; 412 | 231; 321; 412 | 231; 321; 412 |
| Shrubland | 322; 323; 324 | 322; 323; 324 | 322; 323; 324 | 322; 323; 324 | 322; 323; 324 |
| Bare soil | 331; 332; 333; 334; 335 | 331; 332; 333; 334; 335 | 331; 332; 333; 334; 335 | 331; 332; 333; 334; 335 | 331; 332; 333; 334; 335 |
| Water | 411; 421; 422; 423; 511; 512; 521; | 411; 421; 422; 423; 511; 512; 521; | 411; 421; 422; 423; 511; 512; 521; | 411; 421; 422; 423; 511; 512; 521; | 411; 421; 422; 423; 511; 512; 521; |
|  | 522; 523 | 522; 523 | 522; 523 | 522; 523 | 522; 523 |

**Table S6** Land cover category homogenization of SIOSE to the categories defined on the Supervised Classification (SC). In brackets the name of the field used in each SIOSE map to define land cover.

| **SC Category** | **SIOSE Category 2005-2014 (CODIIGE)** | **SIOSE Category 2017 (ID_COBERTU)** |
| --- | --- | --- |
| Urban | 100; 101; 102; 104; 111; 121; 131; | 100; 101; 102; 104; 111; 121; 131; |
|  | 122; 123; 130; 172; 112; 171; 113; | 122; 123; 130; 172; 112; 171; 113; |
|  | 161; 162;140 | 161; 162; 163; 140; 142; 133; 145; |
|  |  | 141; 143; 144; 132 |
| Water | 103; 400; 410; 411; 412; 413; 420; 421; | 103; 400; 410; 411; 412; 413; 420; 421; |
|  | 422; 500; 510; 511; 512; 513; 514; 515; | 422; 500; 510; 511; 512; 513; 514; 515; |
|  | 520; 521; 522; 523; 414 | 520; 521; 522; 523; 414; 423; 105; 106 |
| Agriculture | 200; 210; 211; 212; 220; 221; 222; 223; | 200; 210; 211; 212; 220; 221; 222; 223; |
|  | 231; 232; 241, 150; 250; 260; 233; 235 | 231; 232; 241, 150; 250; 260; 233; 235; |
|  |  | 280; 224; 256; 251 |
| Grassland | 290; 300; 114; 240 | 290; 300; 114; 240; 302 |
| Natural forest | 310; 311; 312; 313 | 310; 311; 312; 313 |
| Plantation forest | 316 | 316 |
| Shrubland | 320; 340 | 320; 340; 301 |
| Bare soil | 330; 331; 333; 334; 335; 336; 350; 351; | 330; 331; 333; 334; 335; 336; 350; 351; |
|  | 352; 353; 354 | 352; 353; 354 |

**Table S7** Land cover category homogenization of NFI to the categories defined on the Supervised Classification (SC). In brackets the name of the field used in each NFI map to define land cover.

| **SC Category** | **NFI Category 2005 (DES_TIPEST)** | **NFI Category 2010-2016 (DES_TIPEST)** | **NFI Category 2022 (TIPES_es)** |
| --- | --- | --- | --- |
| Bare Soil | Afloramientos rocosos | Afloramientos rocosos | Afloramientos rocosos |
|  | Acantilados marinos | Acantilados marinos | Acantilados marinos |
|  | Canchales | Canchales | Playas, dunas y arenales |
|  | Cárcavas | Cárcavas | Canchales |
|  | Playas, dunas y arenales | Playas, dunas y arenales | Cárcavas |
|  | Monte sin Veg. Superior |  | Salinas continentales |
|  | Roquedos con pastoreo |  |  |
| Water | Cursos de agua | Cursos de agua | Cursos de agua |
|  | Lagunas | Lagunas | Estuarios |
|  | Pantano, embalse | Pantano, embalse | Lagunas |
|  | Mares y océanos | Mares y océanos | Mares y océanos |
|  | Zonas pantanosas | Marismas | Marismas |
|  | Agua | Estuarios | Pantano, embalse |
|  | Humedal |  | Zonas pantanosas |
|  | Mar |  |  |
| Shrubland | Arbustedos | Arbustedos | Arbustedos |
|  | Pastizal-Matorral | Pastizal-Matorral | Pastizal-Matorral |
|  | Pastizal-matorral |  |  |
|  | Matorral |  |  |
| Natural forest | Bosque | Bosque | Bosque |
|  | Bosque de Galería | Bosque de Galería | Bosque de Galería |
|  | Complementos Bosque |  |  |
|  | Arboles fuera del monte- A.F.M.(Riberas) |  |  |
|  | Riberas en zona forestal |  |  |
| Agriculture | Cultivos | Cultivos | Cultivos |
|  | Primario | Primario | Primario |
|  | Agrícola | Mosaico agrícola con artificial | Mosaico agrícola con artificial |
| Plantation forest | Bosque de Plantación | Bosque de Plantación | Bosque de Plantación |
| Grassland | Herbazal-Pastizal | Herbazal-Pastizal | Herbazal-Pastizal |
|  | Prados | Prados | Prados |
|  | Prados con setos | Prados con setos | Prados con setos |
|  | Prado | Turberas | Turberas |
|  | Prado con setos/ con sebes |  |  |
|  | Herbazal |  |  |
| Urban | Rest of categories | Rest of categories | Rest of categories |

**Table S8** Number of training and validation points migrated from the original reference point set (manually selected from a 2016 summer image) to each period, along with the number of Landsat images used.

| **Period** | **Landsat Images** | **Training Points** | **Validation Points** | **Total Reference Points** |
| --- | --- | --- | --- | --- |
| 1984–1989 | 136 | 634 | 295 | 930 |
| 1990–1994 | 158 | 642 | 285 | 928 |
| 1995–1999 | 153 | 683 | 278 | 961 |
| 2000–2004 | 206 | 640 | 321 | 962 |
| 2005–2009 | 184 | 666 | 303 | 969 |
| 2010–2014 | 210 | 729 | 279 | 1009 |
| 2015–2018 | 262 | 765 | 273 | 1038 |
| 2019–2023 | 287 | 737 | 280 | 1017 |
| **All periods (1984-2023)** | **1,596** | **5,496** | **2,314** | **7,814** |

**Table S9** User’s and producer’s accuracy (%) by periods for each of the land cover categories: Agriculture (AGR), bare soil (BS), grassland (GRS), natural forest (NF), plantation forest (PF), Shrubland (SHR), urban (URB) and water (WAT).

|  | **Period** | **AGR** | **BS** | **GRS** | **NF** | **PF** | **SHR** | **URB** | **WAT** |
| --- | --- | --- | --- | --- | --- | --- | --- | --- | --- |
| User's accuracy | 1984-1989 | 89.5 | 81.8 | 71.1 | 91.7 | 83.3 | 51.5 | 82.8 | 100.0 |
|  | 1990-1994 | 95.2 | 68.4 | 75.0 | 88.3 | 83.3 | 79.3 | 82.4 | 100.0 |
|  | 1995-1999 | 94.7 | 92.0 | 72.2 | 89.2 | 88.1 | 66.7 | 94.1 | 100.0 |
|  | 2000-2004 | 100.0 | 90.0 | 85.7 | 89.2 | 90.1 | 89.5 | 91.9 | 100.0 |
|  | 2005-2009 | 88.9 | 91.7 | 84.4 | 96.2 | 93.2 | 88.2 | 91.7 | 100.0 |
|  | 2010-2014 | 88.9 | 100.0 | 90.2 | 88.9 | 90.0 | 85.4 | 96.3 | 100.0 |
|  | 2015-2018 | 89.7 | 92.0 | 87.5 | 94.9 | 86.0 | 90.0 | 100.0 | 100.0 |
|  | 2019-2023 | 93.8 | 66.7 | 85.2 | 94.3 | 93.2 | 75.0 | 100.0 | 100.0 |
| Producer's accuracy | 1984-1989 | 89.5 | 81.8 | 71.1 | 91.7 | 83.3 | 51.5 | 82.8 | 100.0 |
|  | 1990-1994 | 95.2 | 68.4 | 75.0 | 88.3 | 83.3 | 79.3 | 82.4 | 100.0 |
|  | 1995-1999 | 94.7 | 92.0 | 72.2 | 89.2 | 88.1 | 66.7 | 94.1 | 100.0 |
|  | 2000-2004 | 100.0 | 90.0 | 85.7 | 89.2 | 90.1 | 89.5 | 91.9 | 100.0 |
|  | 2005-2009 | 88.9 | 91.7 | 84.4 | 96.2 | 93.2 | 88.2 | 91.7 | 100.0 |
|  | 2010-2014 | 88.9 | 100.0 | 90.2 | 88.9 | 90.0 | 85.4 | 96.3 | 100.0 |
|  | 2015-2018 | 89.7 | 92.0 | 87.5 | 94.9 | 86.0 | 90.0 | 100.0 | 100.0 |
|  | 2019-2023 | 93.8 | 66.7 | 85.2 | 94.3 | 93.2 | 75.0 | 100.0 | 100.0 |

**Table S10** Confusion matrix of all the assessed validation points of all periods. Reference category is the one that was manually given to the validation points. Prediction category is the one given by the SC. User's accuracy (UA), producer's accuracy (PA), commission error (CE), omission error (OE), and overall accuracy (OA) are also detailed aside from the confusion matrix. Agriculture (AGR), bare soil (BS), grassland (GRS), natural forest (NF), plantation forest (PF), Shrubland (SHR), urban (URB) and water (WAT).

| **Reference** | **AGR** | **BS** | **GRS** | **NF** | **PF** | **SHR** | **URB** | **WAT** | **UA** | **CE** |
| --- | --- | --- | --- | --- | --- | --- | --- | --- | --- | --- |
| **Prediction** |  |  |  |  |  |  |  |  |  |  |
| **AGR** | 141 | 4 | 4 | 0 | 0 | 1 | 6 | 0 | 90.4 | 9.6 |
| **BS** | 5 | 154 | 0 | 0 | 0 | 1 | 6 | 0 | 92.8 | 7.2 |
| **GRS** | 4 | 0 | 237 | 1 | 11 | 27 | 5 | 0 | 83.2 | 16.8 |
| **NF** | 0 | 0 | 1 | 513 | 30 | 9 | 0 | 0 | 92.8 | 7.2 |
| **PF** | 0 | 0 | 12 | 42 | 481 | 22 | 0 | 0 | 86.4 | 13.6 |
| **SHR** | 2 | 3 | 38 | 4 | 22 | 217 | 3 | 0 | 75.1 | 24.9 |
| **URB** | 1 | 18 | 0 | 0 | 0 | 0 | 239 | 0 | 92.6 | 7.4 |
| **WAT** | 0 | 0 | 0 | 0 | 0 | 0 | 0 | 50 | 100 | 0 |
| **PA** | 92.2 | 86.0 | 81.2 | 91.6 | 88.4 | 78.3 | 92.3 | 100 | **OA =** | **87.8%** |
| **OE** | 7.8 | 14.0 | 18.8 | 8.4 | 11.6 | 21.7 | 7.7 | 0 |  |  |

**Table S11** Estimated total area (in hectareas) for each Land Cover category in each period at the Catchment scale. Agriculture (AGR), bare soil (BS), grassland (GRS), natural forest (NF), plantation forest (PF), Shrubland (SHR), urban (URB) and water (WAT).

| **Park** | **Land cover** | **1984-1989** | **1990-1994** | **1995-1999** | **2000-2004** | **2005-2009** | **2010-2014** | **2015-2018** | **2019-2023** |
| --- | --- | --- | --- | --- | --- | --- | --- | --- | --- |
| **Aizkorri** | **AGR** | 33.29 | 74.67 | 22.13 | 26.16 | 50.71 | 6.64 | 0.89 | 2.23 |
|  | **BS** | 104.14 | 106.72 | 122.44 | 112.46 | 107.47 | 94.32 | 103.29 | 101.43 |
|  | **GRS** | 1205.89 | 1033.27 | 1161.97 | 1014.39 | 1074.34 | 1021.92 | 971.03 | 1046.82 |
|  | **NF** | 2027.08 | 2035.05 | 1920.31 | 2100.61 | 1888.26 | 2058.26 | 2176.57 | 2129.48 |
|  | **PF** | 1499.34 | 1470.57 | 1495.48 | 1326.91 | 1565.83 | 1526.91 | 1387.88 | 1514.33 |
|  | **SHR** | 477.55 | 632.82 | 628.04 | 771.82 | 670.78 | 645.33 | 712.09 | 555.42 |
|  | **URB** | 7.07 | 4.96 | 10.04 | 8.06 | 2.96 | 7.31 | 8.88 | 10.97 |
|  | **WAT** | 2.83 | 0.46 | 0.26 | 0.26 | 0.2 | 0 | 0 | 0 |
| **Aralar** | **AGR** | 32.06 | 102.87 | 51.73 | 73.94 | 107.35 | 10.24 | 0.76 | 12.3 |
|  | **BS** | 125.1 | 83.32 | 124.81 | 107.83 | 127.29 | 102 | 95.65 | 111.11 |
|  | **GRS** | 1513.86 | 1243.72 | 1245.71 | 1029.38 | 1089.35 | 1130.2 | 993.85 | 1025.58 |
|  | **NF** | 3223.42 | 3287.85 | 3038.34 | 3157.17 | 3079.95 | 3229.23 | 3429.18 | 3468.89 |
|  | **PF** | 1576.49 | 1574.56 | 1839.22 | 1692.02 | 1816.22 | 1818.8 | 1643.94 | 1602.12 |
|  | **SHR** | 457.86 | 616.86 | 619.81 | 859.66 | 705.5 | 631.9 | 754.42 | 701.24 |
|  | **URB** | 17.11 | 14.01 | 17.21 | 16.44 | 10.06 | 13.94 | 18.84 | 16.14 |
|  | **WAT** | 1.32 | 15.66 | 15.01 | 15.53 | 15.86 | 15.6 | 15.07 | 14.61 |
| **Artikutza** | **AGR** | 0 | 0.52 | 0.2 | 0 | 0 | 0.13 | 0 | 0 |
|  | **BS** | 0.39 | 0.39 | 0 | 0.46 | 0.26 | 0.46 | 0.79 | 1.64 |
|  | **GRS** | 94.92 | 131.13 | 142.63 | 96.67 | 121.32 | 92.3 | 102.96 | 137.32 |
|  | **NF** | 3352.96 | 3375.89 | 3249.04 | 3202.65 | 3309 | 3408.18 | 3265.42 | 3364.5 |
|  | **PF** | 913.9 | 867.64 | 929.13 | 810.95 | 665.22 | 590.5 | 617.55 | 635.29 |
|  | **SHR** | 334.72 | 322.06 | 376.44 | 587.23 | 601.31 | 606 | 710.99 | 568.19 |
|  | **URB** | 0.22 | 0.2 | 0.46 | 0.33 | 0.2 | 0.26 | 1.11 | 1.97 |
|  | **WAT** | 11.41 | 10.69 | 11.01 | 10.62 | 11.54 | 11.08 | 10.1 | 0 |
| **Gorbea** | **AGR** | 27.72 | 38.04 | 6.38 | 6.81 | 5.35 | 2.88 | 1.32 | 0.63 |
|  | **BS** | 50.63 | 47.08 | 75.39 | 59.21 | 86.9 | 68.37 | 64.25 | 72.06 |
|  | **GRS** | 550.71 | 371.23 | 445.93 | 406.56 | 539.9 | 553.14 | 517.62 | 367.94 |
|  | **NF** | 1481.62 | 1547.18 | 1447.48 | 1410.2 | 1400.69 | 1418.09 | 1428.08 | 1196.02 |
|  | **PF** | 1005.44 | 942.43 | 926.73 | 810.92 | 815.01 | 830.59 | 847.11 | 892.81 |
|  | **SHR** | 764.46 | 970.76 | 1013.95 | 1220.97 | 1067.22 | 1030.68 | 1056.47 | 1387.59 |
|  | **URB** | 0.78 | 0.26 | 1.25 | 0.66 | 0 | 0.07 | 0 | 0.07 |
|  | **WAT** | 0 | 0 | 0 | 0 | 0 | 0 | 0 | 0 |
| **Izki** | **AGR** | 149.65 | 200.22 | 144.89 | 143.85 | 159.66 | 126.08 | 139.65 | 130.28 |
|  | **BS** | 11.76 | 21.01 | 14.25 | 18.03 | 12.87 | 20.3 | 25.84 | 27.28 |
|  | **GRS** | 360.33 | 107.85 | 206.69 | 154.45 | 188.43 | 127.16 | 116.72 | 157.83 |
|  | **NF** | 3004.67 | 3152.24 | 3237.5 | 3148.13 | 3316.99 | 3348.46 | 3292.77 | 3362.41 |
|  | **PF** | 370.83 | 264.96 | 309.92 | 290.17 | 297.05 | 287.6 | 263.88 | 300.73 |
|  | **SHR** | 454.74 | 610.86 | 436.11 | 593.76 | 382.39 | 450.08 | 517.89 | 380.74 |
|  | **URB** | 7.94 | 1.65 | 10.57 | 11.55 | 2.41 | 0.24 | 3.17 | 0.65 |
|  | **WAT** | 0 | 0 | 0 | 0 | 0.13 | 0 | 0 | 0 |

**Table S12** Estimated total area (in hectareas) for each Land Cover category in each period at the Riparian scale. Agriculture (AGR), bare soil (BS), grassland (GRS), natural forest (NF), plantation forest (PF), Shrubland (SHR), urban (URB) and water (WAT).

| **Park** | **Land cover** | **1984-1989** | **1990-1994** | **1995-1999** | **2000-2004** | **2005-2009** | **2010-2014** | **2015-2018** | **2019-2023** |
| --- | --- | --- | --- | --- | --- | --- | --- | --- | --- |
| **Aizkorri** | **AGR** | 0.36 | 0.91 | 0.01 | 0 | 0 | 0 | 0 | 0 |
|  | **BS** | 0.29 | 0.45 | 0.3 | 0 | 0.11 | 0.24 | 0.32 | 0.05 |
|  | **GRS** | 22.3 | 16.41 | 14 | 8.02 | 10.84 | 7.68 | 6.13 | 8.24 |
|  | **NF** | 82.48 | 92.09 | 84.97 | 103.13 | 86.93 | 99.95 | 105.59 | 105.73 |
|  | **PF** | 53.62 | 47.24 | 58.33 | 46.47 | 58.69 | 48.77 | 41.1 | 46.3 |
|  | **SHR** | 9.07 | 12.68 | 12.01 | 12.51 | 13.79 | 13.75 | 17.19 | 9.9 |
|  | **URB** | 0.76 | 0.42 | 0.67 | 0.23 | 0 | 0.17 | 0.23 | 0.33 |
|  | **WAT** | 1.68 | 0.35 | 0.26 | 0.21 | 0.2 | 0 | 0 | 0 |
| **Aralar** | **AGR** | 0.33 | 0.69 | 0.24 | 0.58 | 0.18 | 0.16 | 0 | 0.16 |
|  | **BS** | 0.25 | 0.44 | 0.85 | 0.32 | 1.73 | 1.03 | 0.74 | 2.3 |
|  | **GRS** | 63.47 | 51.83 | 50.16 | 34.92 | 47.12 | 42.77 | 31.46 | 33.13 |
|  | **NF** | 101.31 | 110.15 | 93.06 | 100.82 | 96 | 107.56 | 122.61 | 126.23 |
|  | **PF** | 37.33 | 33.55 | 54.19 | 48.78 | 47.88 | 40.59 | 29.6 | 31.65 |
|  | **SHR** | 19.91 | 26.31 | 24.47 | 37.95 | 32.7 | 32.48 | 39.46 | 31.03 |
|  | **URB** | 6.37 | 5.59 | 6.42 | 6.03 | 3.78 | 4.79 | 5.53 | 4.88 |
|  | **WAT** | 0.42 | 0 | 0 | 0 | 0 | 0 | 0 | 0 |
| **Artikutza** | **AGR** | 0 | 0.07 | 0 | 0 | 0 | 0 | 0 | 0 |
|  | **BS** | 0 | 0 | 0 | 0 | 0 | 0.05 | 0.22 | 1.15 |
|  | **GRS** | 1.35 | 0.99 | 0.88 | 0.9 | 1.28 | 0.61 | 0.7 | 0.69 |
|  | **NF** | 163.27 | 172.74 | 170.24 | 167.37 | 176.01 | 177.02 | 174.54 | 178.67 |
|  | **PF** | 24.3 | 15.37 | 17.34 | 15.03 | 6.5 | 4.94 | 5.96 | 9.26 |
|  | **SHR** | 3.74 | 3.65 | 3.87 | 9.37 | 8.64 | 9.84 | 11.24 | 7.97 |
|  | **URB** | 0.15 | 0.15 | 0.34 | 0.15 | 0.15 | 0.22 | 0.52 | 1.11 |
|  | **WAT** | 6.04 | 5.89 | 6.19 | 6.02 | 6.27 | 6.15 | 5.67 | 0 |
| **Gorbea** | **AGR** | 2.31 | 1.18 | 0 | 0.2 | 0 | 0 | 0 | 0 |
|  | **BS** | 0.87 | 1.03 | 2.28 | 1.64 | 2.65 | 2.19 | 1.9 | 2.19 |
|  | **GRS** | 18.79 | 6.28 | 6.3 | 4.63 | 8.34 | 4.65 | 6.65 | 3.54 |
|  | **NF** | 53.03 | 65.77 | 58.15 | 53.74 | 57.41 | 59.34 | 58.97 | 40.49 |
|  | **PF** | 20.27 | 14.21 | 15.39 | 7.46 | 6.84 | 7.75 | 9.04 | 15.9 |
|  | **SHR** | 27.25 | 34.05 | 40.41 | 54.86 | 47.28 | 48.59 | 45.97 | 60.4 |
|  | **URB** | 0 | 0 | 0 | 0 | 0 | 0 | 0 | 0 |
|  | **WAT** | 0 | 0 | 0 | 0 | 0 | 0 | 0 | 0 |
| **Izki** | **AGR** | 2.84 | 5.48 | 2.31 | 3.82 | 3.44 | 1.55 | 1.62 | 2.73 |
|  | **BS** | 0 | 0 | 0 | 0 | 0 | 0 | 0 | 0 |
|  | **GRS** | 20.15 | 3.41 | 10.06 | 5.35 | 6.29 | 3.88 | 2.51 | 3.94 |
|  | **NF** | 150.25 | 161.3 | 169.52 | 163.05 | 174.65 | 175.07 | 175.56 | 177.86 |
|  | **PF** | 16.68 | 6.64 | 7.01 | 3.96 | 4.27 | 4.44 | 4.65 | 5.07 |
|  | **SHR** | 18.86 | 32.04 | 20.08 | 32.81 | 20.33 | 24.06 | 24.66 | 19.4 |
|  | **URB** | 0.21 | 0.06 | 0.02 | 0 | 0 | 0 | 0 | 0 |
|  | **WAT** | 0 | 0 | 0 | 0 | 0 | 0 | 0 | 0 |

**Table S13** Estimated total area (in hectareas) for each Land Cover category in each period at the Reach scale. Agriculture (AGR), bare soil (BS), grassland (GRS), natural forest (NF), plantation forest (PF), Shrubland (SHR), urban (URB) and water (WAT).

| **Park** | **Land cover** | **1984-1989** | **1990-1994** | **1995-1999** | **2000-2004** | **2005-2009** | **2010-2014** | **2015-2018** | **2019-2023** |
| --- | --- | --- | --- | --- | --- | --- | --- | --- | --- |
| **Aizkorri** | **AGR** | 0 | 0 | 0 | 0 | 0.06 | 0 | 0 | 0 |
|  | **BS** | 0 | 0 | 0 | 0 | 0 | 0 | 0.07 | 0 |
|  | **GRS** | 5.64 | 5.01 | 5.69 | 4.28 | 6.08 | 3.92 | 4.32 | 5.79 |
|  | **NF** | 8.77 | 8.79 | 8.1 | 11.05 | 9.3 | 11.56 | 12.48 | 13.37 |
|  | **PF** | 15.83 | 15.83 | 14.46 | 13.09 | 11.58 | 11.81 | 8.67 | 8.96 |
|  | **SHR** | 0.69 | 1.3 | 2.68 | 2.5 | 3.87 | 3.64 | 5.39 | 2.68 |
|  | **URB** | 0 | 0 | 0 | 0 | 0.03 | 0 | 0 | 0.13 |
|  | **WAT** | 0 | 0 | 0 | 0 | 0 | 0 | 0 | 0 |
| **Aralar** | **AGR** | 0 | 0 | 0.24 | 0.18 | 0.05 | 0.21 | 0.11 | 0 |
|  | **BS** | 0 | 0.08 | 0 | 0.02 | 0.09 | 0.16 | 0.07 | 0.32 |
|  | **GRS** | 8.76 | 7.3 | 6.38 | 4.14 | 5.18 | 4.32 | 3.39 | 3.11 |
|  | **NF** | 10.54 | 11.46 | 10.25 | 10.93 | 10.23 | 11.59 | 12.94 | 13.33 |
|  | **PF** | 6.77 | 5.38 | 8.04 | 7.46 | 8.89 | 7.96 | 6.16 | 6.63 |
|  | **SHR** | 4.6 | 6.34 | 5.27 | 6.81 | 5.32 | 5.46 | 6.77 | 6.24 |
|  | **URB** | 0.25 | 0.37 | 0.74 | 1.37 | 1.16 | 1.23 | 1.47 | 1.29 |
|  | **WAT** | 0 | 0 | 0 | 0 | 0 | 0 | 0 | 0 |
| **Artikutza** | **AGR** | 0 | 0 | 0 | 0 | 0 | 0 | 0 | 0 |
|  | **BS** | 0 | 0 | 0 | 0 | 0 | 0 | 0 | 0 |
|  | **GRS** | 0.22 | 0.93 | 0.96 | 0.79 | 0.67 | 1.03 | 0.52 | 0.67 |
|  | **NF** | 24.67 | 27.08 | 26.2 | 25.11 | 26.28 | 27.37 | 26.12 | 26.3 |
|  | **PF** | 5.56 | 2.78 | 3.41 | 2.92 | 3.03 | 1.58 | 2.46 | 3.07 |
|  | **SHR** | 0.47 | 0.13 | 0.35 | 2.1 | 0.94 | 0.94 | 1.82 | 0.89 |
|  | **URB** | 0 | 0 | 0 | 0 | 0 | 0 | 0 | 0 |
|  | **WAT** | 0 | 0 | 0 | 0 | 0 | 0 | 0 | 0 |
| **Gorbea** | **AGR** | 0 | 0.18 | 0.17 | 0.2 | 0.26 | 0.17 | 0.23 | 0.08 |
|  | **BS** | 0 | 0 | 0 | 0 | 0 | 0.06 | 0 | 0 |
|  | **GRS** | 5.05 | 2.33 | 1.7 | 1.33 | 2.58 | 1.1 | 1.3 | 1.1 |
|  | **NF** | 10.43 | 12.22 | 13.35 | 12.64 | 12.03 | 12.26 | 12.51 | 6.56 |
|  | **PF** | 8.7 | 6.37 | 4.91 | 2.61 | 2.55 | 3.13 | 3.64 | 5.81 |
|  | **SHR** | 6.75 | 9.82 | 10.64 | 14.09 | 13.51 | 14.2 | 13.24 | 17.37 |
|  | **URB** | 0 | 0 | 0.15 | 0.06 | 0 | 0 | 0 | 0 |
|  | **WAT** | 0 | 0 | 0 | 0 | 0 | 0 | 0 | 0 |
| **Izki** | **AGR** | 1.63 | 2 | 1.24 | 0.84 | 1.88 | 1.17 | 1.12 | 0.91 |
|  | **BS** | 0 | 0 | 0 | 0 | 0 | 0 | 0 | 0.07 |
|  | **GRS** | 3.49 | 0.21 | 1.93 | 1.25 | 1.59 | 0.74 | 0.54 | 1.07 |
|  | **NF** | 15.23 | 16.43 | 19.02 | 17.99 | 19.77 | 20.46 | 20.06 | 21.12 |
|  | **PF** | 2.51 | 1.77 | 2.19 | 2.08 | 2.04 | 2.09 | 2.43 | 2.85 |
|  | **SHR** | 8.07 | 10.5 | 6.55 | 8.76 | 5.65 | 6.47 | 6.77 | 4.91 |
|  | **URB** | 0 | 0 | 0 | 0 | 0 | 0 | 0 | 0 |
|  | **WAT** | 0 | 0 | 0 | 0 | 0 | 0 | 0 | 0 |

**Table S14** Initial (1987) and final (2021) land cover percentages estimated by SC of each land cover type (LC)- Agriculture (AGR), bare soil (BS), grassland (GRS), natural forest (NF), plantation forest (PF), Shrubland (SHR), urban (URB) and water (WAT)- in the 5 protected areas at the 3 scales - Reach (Re), Riparian (Ri), Catchment (Ca). Only the slopes of significant linear models are shown.

|  |  | **Aizkorri** | | | **Aralar** | | | **Artikutza** | | | **Gorbea** | | | **Izki** | | |
| --- | --- | --- | --- | --- | --- | --- | --- | --- | --- | --- | --- | --- | --- | --- | --- | --- |
| **LC** | **Scale** | **1987** | **2021** | **Slope** | **1987** | **2021** | **Slope** | **1987** | **2021** | **Slope** | **1987** | **2021** | **Slope** | **1987** | **2021** | **Slope** |
| **AGR** | Re | 0.00% | 0.00% |  | 0.00% | 0.00% |  | 0.00% | 0.00% |  | 0.00% | 0.26% |  | 5.27% | 2.96% |  |
|  | Ri | 0.21% | 0.00% |  | 0.15% | 0.07% |  | 0.00% | 0.00% |  | 1.89% | 0.00% | -0.044 | 1.36% | 1.30% |  |
|  | Ca | 0.62% | 0.04% |  | 0.46% | 0.18% |  | 0.00% | 0.00% |  | 0.71% | 0.02% | -0.024 | 3.43% | 2.99% |  |
| **BS** | Re | 0.00% | 0.00% |  | 0.00% | 1.05% | 0.021 | 0.00% | 0.00% |  | 0.00% | 0.00% |  | 0.00% | 0.21% |  |
|  | Ri | 0.17% | 0.03% |  | 0.11% | 1.00% | 0.019 | 0.00% | 0.58% |  | 0.71% | 1.79% |  | 0.00% | 0.00% |  |
|  | Ca | 1.94% | 1.89% |  | 1.80% | 1.60% |  | 0.01% | 0.03% |  | 1.30% | 1.84% |  | 0.27% | 0.63% | 0.008 |
| **GRS** | Re | 18.23% | 18.71% |  | 28.34% | 10.05% | -0.504 | 0.73% | 2.16% |  | 16.32% | 3.57% | -0.262 | 11.28% | 3.45% |  |
|  | Ri | 13.07% | 4.83% | -0.237 | 27.67% | 14.44% | -0.344 | 0.68% | 0.35% |  | 15.33% | 2.89% |  | 9.64% | 1.89% |  |
|  | Ca | 22.51% | 19.53% |  | 21.79% | 14.75% | -0.174 | 2.02% | 2.92% |  | 14.19% | 9.39% |  | 8.26% | 3.62% |  |
| **NF** | Re | 28.35% | 43.22% | 0.462 | 34.07% | 43.11% | 0.235 | 79.79% | 85.04% |  | 33.72% | 21.20% |  | 49.24% | 68.29% | 0.514 |
|  | Ri | 48.36% | 61.99% | 0.367 | 44.16% | 55.03% |  | 82.11% | 89.85% | 0.177 | 43.28% | 33.05% |  | 71.89% | 85.10% | 0.340 |
|  | Ca | 37.84% | 39.72% |  | 46.40% | 49.90% |  | 71.21% | 71.45% |  | 38.17% | 30.53% | -0.168 | 68.92% | 77.12% | 0.206 |
| **PF** | Re | 51.20% | 28.97% | -0.732 | 21.91% | 21.43% |  | 17.97% | 9.92% |  | 28.13% | 18.80% |  | 8.11% | 9.22% |  |
|  | Ri | 31.44% | 27.14% |  | 16.27% | 13.80% |  | 12.22% | 4.65% | -0.244 | 16.54% | 12.98% |  | 7.98% | 2.42% |  |
|  | Ca | 27.99% | 28.25% |  | 22.69% | 23.05% |  | 19.41% | 13.49% | -0.226 | 25.90% | 22.79% |  | 8.51% | 6.90% |  |
| **SHR** | Re | 2.22% | 8.67% | 0.308 | 14.87% | 20.18% |  | 1.51% | 2.88% |  | 21.84% | 56.17% | 0.792 | 26.10% | 15.86% | -0.344 |
|  | Ri | 5.32% | 5.81% |  | 8.68% | 13.53% | 0.174 | 1.88% | 4.01% | 0.105 | 22.24% | 49.30% | 0.607 | 9.02% | 9.28% |  |
|  | Ca | 8.91% | 10.36% |  | 6.59% | 10.09% |  | 7.11% | 12.07% | 0.223 | 19.70% | 35.42% | 0.284 | 10.43% | 8.73% |  |
| **URB** | Re | 0.00% | 0.43% |  | 0.82% | 4.18% | 0.111 | 0.00% | 0.00% |  | 0.00% | 0.00% |  | 0.00% | 0.00% |  |
|  | Ri | 0.44% | 0.20% |  | 2.78% | 2.13% |  | 0.08% | 0.56% |  | 0.00% | 0.00% |  | 0.10% | 0.00% | -0.002 |
|  | Ca | 0.13% | 0.20% |  | 0.25% | 0.23% |  | 0.00% | 0.04% | 0.001 | 0.02% | 0.00% |  | 0.18% | 0.01% |  |
| **WAT** | Re | 0.00% | 0.00% |  | 0.00% | 0.00% |  | 0.00% | 0.00% |  | 0.00% | 0.00% |  | 0.00% | 0.00% |  |
|  | Ri | 0.99% | 0.00% | -0.021 | 0.18% | 0.00% |  | 3.04% | 0.00% |  | 0.00% | 0.00% |  | 0.00% | 0.00% |  |
|  | Ca | 0.05% | 0.00% | -0.001 | 0.02% | 0.21% |  | 0.24% | 0.00% |  | 0.00% | 0.00% |  | 0.00% | 0.00% |  |

**Table S15** F-statistics and p-values of comparison between linear models.

|  | **Scale** | **Map** | **Intercept** | **Slope** | **F-statistic** | ***p-value*** |
| --- | --- | --- | --- | --- | --- | --- |
| **Land Cover area** | **Catchment** | **CORINE** | 3.91E-03 | 0.95 | 0.47 | 0.624 |
|  |  | **SIOSE** | -0.012 | 1.10 | 1.82 | 0.167 |
|  |  | **NFI** | 0.032 | 0.69 | 17.12 | <0.001 |
|  | **Riparian** | **CORINE** | 6.88E-03 | 0.95 | 2.33 | 0.100 |
|  |  | **SIOSE** | -5.49E-03 | 1.04 | 0.54 | 0.585 |
|  |  | **NFI** | 2.78E-03 | 0.98 | 0.42 | 0.656 |
|  | **Reach** | **CORINE** | 0.193 | 1.14 | 58.91 | <0.001 |
|  |  | **SIOSE** | 0.108 | 0.88 | 17.51 | <0.001 |
|  |  | **NFI** | 0.125 | 0.62 | 26.83 | <0.001 |
| **Land Cover Trend** | **Catchment** | **CORINE** | -1.75E-04 | -2.16 | 6.49 | 0.004 |
|  |  | **SIOSE** | -1.95E-03 | 0.76 | 3.23 | 0.050 |
|  |  | **NFI** | 1.22E-03 | -0.620 | 1.58 | 0.219 |
|  | **Riparian** | **CORINE** | -5.11E-14 | 0.59 | 1.26 | 0.296 |
|  |  | **SIOSE** | -6.78E-04 | 0.31 | 6.97 | 0.003 |
|  |  | **NFI** | 1.00E-07 | 0.14 | 109.30 | <0.001 |
|  | **Reach** | **CORINE** | 3.88E-04 | 1.02 | 0.05 | 0.947 |
|  |  | **SIOSE** | -5.55E-03 | 1.11 | 4.16 | 0.023 |
|  |  | **NFI** | -4.04E-04 | -0.170 | 5.79 | 0.006 |

**Figure S1** Land cover maps from all four methods for the most recent period with complete coverage (2015–2018), shown for each catchment (top to bottom: Artikutza, Gorbea, Aizkorri, Aralar, Izki).
